# Computational and Bioinformatics Techniques for Immunology

**DOI:** 10.1155/2014/263189

**Published:** 2014-12-31

**Authors:** Francesco Pappalardo, Vladimir Brusic, Filippo Castiglione, Christian Schönbach

**Affiliations:** ^1^Department of Drug Sciences, University of Catania, 95125 Catania, Italy; ^2^School of Science and Technology, Nazarbayev University, Astana 010000, Kazakhstan; ^3^Istituto Applicazioni del Calcolo “M. Picone”, Consiglio Nazionale delle Ricerche, Via dei Taurini 19, 00185 Rome, Italy

Computational immunology and immunological bioinformatics are well-established and rapidly evolving research fields. Whereas the former aims to develop mathematical and/or computational methods to study the dynamics of cellular and molecular entities during the immune response [1–4], the latter targets proposing methods to analyze large genomic and proteomic immunological-related datasets and derive (i.e., predict) new knowledge mainly by statistical inference and machine learning algorithms.

Since immunology provides key information about basic mechanisms in a number of related diseases, it represents the most critical target for medical intervention. Therefore an advance in either computational or bioinformatics immunology research field has the potential to pave the way for improvement of human health through better patient-specific diagnostics and optimized immune treatment.

In this special issue, we take an interest from mathematicians, bioinformaticians, computational scientists, and engineers together with experimental immunologists, to present and discuss latest developments in different subareas ranging from modeling and simulation to machine learning predictions and their application to basic and clinical immunology.

Of the possible directions for development in immune-informatics special interest is raising for models focusing on innate-adaptive immune response activation, immune senescence, and multiscale and multiorgan models of immune-related diseases and for models accounting for cell trafficking in lymph nodes and/or in the lymphatic mesh as in “*Modeling biology spanning different scales: an open challenge*” by F. Castiglione et al.

Exploring the connections between classical mathematical modeling (at different scales) and bioinformatics predictions of omic scope along with specific aspects of the immune system in combination with concepts and methods like computer simulations, mathematics and statistics for the discovery, design, and optimization of drugs, vaccines, and other immunotherapies represents a hot topic in computational biology and systems medicine [5, 6].

The review from F. Castiglione et al. calls attention to the importance of the different time-space scale involved in biological phenomena and in particular in the immune system. It dissects the problem and discusses various techniques that have been developed in scientific areas other than computational biology.

In their paper S. Jarrah et al. illustrate a simple ODE model to investigate the role of the immune response in muscle degeneration and regeneration in the mdx mouse model of Duchenne muscular dystrophy. Their model suggests that the immune response contributes substantially to the muscle degeneration and regeneration processes and predicts in a certain parameter range a permanent immune activation damaging muscle fibers.

In the paper contributed by T. Clancy and E. Hovig, the authors propose a new method to integrate expression profiles and protein-protein interaction (PPI) data. Bioinformatics techniques are used to study differential protein interaction mechanisms across the entire immune cell lineages and the transcriptional activators and modules and are analyzed in the context of exemplars obtained by clustering the PPI network. The results illustrate that the integration of protein interaction networks with the most comprehensive database of gene expression profiles of the immune cells can be used to generate hypotheses into the underlying mechanisms governing the differentiation and the differential functional activity across the immune cell lineage.

The development of mathematical models of the immune response allows a better understanding of the multifaceted mechanisms of the defense system. In this scenario, as already introduced in the review from F. Castiglione et al., multiscale approaches play a fundamental role. B. de M. Quintela et al. propose a scheme for coupling distinct models of different scales and aspects of the immune system describing a new model that deals with the inflammation processes. These processes are simulated coupling and ordinary differential equations that are used as a model for the systemic response. The dynamics of various immune cells is shown in the presence of an antigen.

There is a controversy about the relationship between HLA-A2 and Alzheimer's disease. HLA supposedly plays a modifier effect on the risk that depends on genetic loadings. Garcia and Murillo present an in silico method to evaluate this relationship and to reveal genes associated with both the HLA-A2 and Alzheimer's disease. They used experimental knowledge of protein-protein interactions to evaluate the top ranked genes shared by both concepts, previously found through text mining.

With the vast amount of immunological data available, immunology research is entering the big data era. These data vary in granularity, quality, and complexity and are stored in various formats, including publications, technical reports, and databases. In the paper contributed by G. L. Zhang et al., it is clearly stated that the present challenge is to make the transition from data to actionable knowledge and wisdom and bridge the gap between knowledge and application. In their work, the authors present a knowledge-based approach based on a framework called KB-builder that facilitates data mining by enabling fast development and deployment of web-accessible immunological data knowledge warehouses. This technique speeds up rational vaccine design by providing accurate and well-annotated data coupled with tailored computational analysis tools and workflows.

Hepatitis C virus and HIV are rapidly mutating viruses. They have adopted evolutionary strategies that allow escape from the host immune response via genomic mutations. Recent advances in high-throughput sequencing are reshaping the field of immune-virology of viral infections, as these allow fast and cheap generation of genomic data. P. Leung et al. propose a pipeline that allows visualization and statistical analysis of viral mutations that are associated with immune escape. Using next generation sequencing data from longitudinal analysis of HCV viral genomes during a single HCV infection, along with antigen specific T-cell responses detected from the same subject, the authors prove the applicability of these tools in the context of primary HCV infection. The proposed pipeline is a freely accessible collection of tools (see the paper for details).

M. Kenn et al. point the attention on the dynamic variations in the distances between pairs of atoms that are used for clustering subdomains of biomolecules. They draw on a well-known target function for clustering and first show mathematically that the assignment of atoms to clusters has to be crisp, not fuzzy, as hitherto assumed, proving that this method reduces the computational load of clustering drastically, demonstrating results for several biomolecules relevant in immunoinformatics.

In the paper by R. Ribarics et al., molecular dynamics is presented as a valuable tool for the investigation of functional elements in biomolecules. They used several spline models to approximate the overall shape of MHC *α*-helices. The authors applied this technique to a series of MD simulations of alloreactive MHC molecules that allowed them to capture the dynamics of MHC *α*-helices' steric configurations. In the paper, they discuss the variability of spline models underlying the geometric analysis with varying polynomial degrees of the splines.

HIV represents a widespread viral infection without cure. Drug treatment has transformed HIV disease into a treatable long-term infection. However, the appearance of mutations within the viral genome reduces the susceptibility of HIV to drugs. In the paper contributed by M. Haering et al., the authors discuss predictions derived from a mathematical model of HIV dynamics. Their results indicate that early therapy initiation (within 2 years after infection) is critical to delay AIDS progression.

*Francesco Pappalardo* *Vladimir Brusic* *Filippo Castiglione* *Christian Schönbach*
